# Gut microbiota modulates visceral sensitivity through calcitonin gene-related peptide (CGRP) production

**DOI:** 10.1080/19490976.2023.2188874

**Published:** 2023-03-20

**Authors:** Julien Pujo, Giada De Palma, Jun Lu, Heather J. Galipeau, Michael G. Surette, Stephen M. Collins, Premysl Bercik

**Affiliations:** Farncombe Family Digestive Health Research Institute, Department of Medicine, McMaster University, Hamilton, Canada

**Keywords:** Visceral pain, bacteria, germ-free, dorsal root ganglia (DRG), substance P (SP), calcitonin-gene-related peptide (CGRP)

## Abstract

Abdominal pain is common in patients with gastrointestinal disorders, but its pathophysiology is unclear, in part due to poor understanding of basic mechanisms underlying visceral sensitivity. Accumulating evidence suggests that gut microbiota is an important determinant of visceral sensitivity. Clinical and basic research studies also show that sex plays a role in pain perception, although the precise pathways are not elucidated. We investigated pain responses in germ-free and conventionally raised mice of both sexes, and assessed visceral sensitivity to colorectal distension, neuronal excitability of dorsal root ganglia (DRG) neurons and the production of substance P and calcitonin gene-related peptide (CGRP) in response to capsaicin or a mixture of G-protein coupled receptor (GPCR) agonists. Germ-free mice displayed greater in vivo responses to colonic distention than conventional mice, with no differences between males and females. Pretreatment with intracolonic capsaicin or GPCR agonists increased responses in conventional, but not in germ-free mice. In DRG neurons, gut microbiota and sex had no effect on neuronal activation by capsaicin or GPCR agonists. While stimulated production of substance P by DRG neurons was similar in germ-free and conventional mice, with no additional effect of sex, the CGRP production was higher in germ-free mice, mainly in females. Absence of gut microbiota increases visceral sensitivity to colorectal distention in both male and female mice. This is, at least in part, due to increased production of CGRP by DRG neurons, which is mainly evident in female mice. However, central mechanisms are also likely involved in this process.

## Introduction

Abdominal pain is common in patients with chronic gastrointestinal disorders including irritable bowel syndrome (IBS) and inflammatory bowel diseases (IBD).^1,2^ Treatment of abdominal pain is challenging, not only because current medications often have undesirable side effects,^3,4^ but also because our understanding of its pathogenesis, as well as basic mechanisms underlying visceral sensitivity, is incomplete.^5^

The basis of pain perception includes the activation of peripheral nerve fibers, which propagate the painful stimuli to the dorsal root ganglia (DRG), transmitting the signal to the dorsal horn of the spinal cord and toward the brain.^6^ Pain sensation occurs when mechanical, heat, and chemical stimuli activate nociceptors present in afferent fibers and propagate the message to the brain.^7,8^ There, pain signals are processed in the primary somatosensory, anterior cingulate and prefrontal cortex, insula, amygdala and thalamus.^7,9^ Pain sensation is modulated by descending pathways, activating an endogenous pain inhibitory system.^7,9^

Pain-mediating neurotransmitters, such as calcitonin gene-related peptide (CGRP) and substance P (SP), are key elements in the transmission of pain from the periphery to the brain.^8,10–12^ These neuropeptides, produced by the DRG neurons, mediate nociceptive signaling to second-order neurons in the spinal cord^12,13^ and co-localize with transient receptor potential vanilloid type 1 (TRPV1) neurons.^13,14^ However, the exact mechanisms involved in their production are still unclear.

Accumulating data suggest that the gut microbiota modulates gut function and interacts with the host nervous system.^15^ Germ-free mice exhibit greater responses to colorectal distension and have lower pain thresholds than conventional mice that normalize after bacterial colonization.^16^ Similarly, antibiotics, by modulation of the gut microbiota, induce visceral hypersensitivity.^17,,18^ Furthermore, several probiotic bacteria were shown to possess anti-nociceptive properties.^19–22^ However, microbiota may have also pro-nociceptive effects as inflammatory pain, induced by carrageenan, was lower in germ-free mice and higher after bacterial colonization.^23^ Bacteria can directly activate nociceptor neurons to produce pain, especially by the production of formyl peptides, α-hemolysin or streptolysin S.^24,25^ On the other hand, some bacterial products such as lipopeptides or anthrax toxins can act on DRG sensory neurons to silence pain.^26,27^

The bacterial modulation of pain appears to be sex-dependent as visceral sensitivity is similar between conventional and germ-free female mice, with ovariectomy inducing visceral hypersensitivity in conventional, but not germ-free mice.^28^ Several animal studies have investigated the sex-specific response to pain perception.^29,30^ Mechanical allodynia after nerve injury is mediated by microglial activation in the spinal cord in male but not in female mice. In contrast, pain in response to nerve injury or inflammation is dependent on adaptive immune cells in females but not in male mice.^30^ A recent study also demonstrated sex differences in visceral pain in the context of acute and persistent colon inflammation.^31^ Accumulating evidence suggests that sex hormones may influence visceral sensitivity^32^ as estrogen facilitated while testosterone attenuated stress-induced visceral hypersensitivity by altering brain-derived neurotrophic factor (BDNF) in the spinal cord.^33,34^ Clinical studies showed that women report more abdominal pain than men,^35^ with IBS female patients being more sensitive to rectal distension than male patients.^36^ The effect of sex on pain perception may be explained by a difference in brain processing of painful stimuli.^37^ However, despite a growing interest in this topic, the exact mechanisms that contribute to sex differences in visceral pain are still not fully elucidated.

In our study, we investigated the effect of gut microbiota on visceral sensitivity *in vivo* using colorectal distension in germ-free and conventional mice and assessed the activity of DRG neurons and their production of SP and CGRP. To probe nociceptive pathways, we have chosen stimulation with capsaicin to activate TRPV1 receptors, and a mixture of agonists (bradykinin, histamine and serotonin) that activate G-protein coupled receptors (GPCR), as they were previously used to investigate the role of microbial metabolites in abdominal pain.^26,,38^ In addition, we explored the effects of female and male sex on visceral sensitivity.

## Methods

### Animals

Female and male mice, raised conventionally with specific pathogen-free (SPF) microbiota, or germ-free C57BL/6 mice, aged to 7–17 weeks old, were used in this study. GF mice were provided by the Axenic Gnotobiotic Unit of McMaster University. The SPF mice were provided by Charles River (Quebec, Canada). Some of the SPF mice were bred in the McMaster Central Animal Facility and their first-generation offsprings (*n* = 22 mice) were used. Mice were housed under 12 hours light/dark cycles and standard conditions for temperature and humidity. SPF and germ-free mice were used to assess visceral sensitivity *in vivo* by colorectal distension (CRD) and to obtain primary cultures of DRG neurons to determine neuronal activity and neurotransmitter production. All experiments were approved by the McMaster University Animal Care Committee.

In this study, 6 cohorts of mice (125 SPF and 91 germ-free mice) were used: 1) total of 31 mice (SPF = 19: 8 females, 11 males; GF = 12: 6 females, 6 males) were subjected to CRD at baseline condition; 2) total of 41 mice (SPF = 29: 12 females, 17 males; GF = 12: 6 females, 6 males) underwent CRD after intracolonic administration of capsaicin (30 µg per animal); 3) total of 34 mice (SPF = 23: 12 females, 11 males; GF = 12: 6 females, 6 males) underwent CRD after intracolonic administration of GPCR agonists (histamine, serotonin, bradykinin; 30 µg per animal); 4) total of 35 mice (SPF = 24: 12 females, 12 males; GF = 11: 5 females, 6 males) received intracolonic administration of vehicle (Tween 80 10%, Ethanol 10% and saline 80%) prior to performing CRD and served as controls for cohorts 2 and 3. The 24 SPF mice that underwent CRD after intracolonic administration of vehicle were submitted after 1 week of rest to CRD in response to intracolonic instillation of capsaicin or GPCR agonists. 5) total of 50 mice (SPF = 30: 16 females, 14 males; GF = 20: 10 females, 10 males) were sacrificed and DRG neurons collected to perform calcium flux imaging; 6) total of 24 mice (SPF = 12: 6 females, 6 males, GF = 12: 6 females, 6 males) were sacrificed and DRG neurons used to assess production of SP; 7) total of 28 mice (SPF = 14: 6 females, 8 males; GF = 12: 6 females, 6 males) were sacrificed and DRG neurons used to assess production of CGRP.

## Colorectal distension and electromyography (EMG) recording

Mice were briefly anaesthetised with isoflurane (Isoflurane USP 99.9%, Fresenius Kabi, Toronto, Canada) and a custom-made catheter balloon (20 mm long x 10 mm wide) was delicately inserted into the colon up to 5 mm from the anus, covering its tip with lubricant, and secured by a tape to mouse tail. Mice were positioned in a custom-made jacket containing two EMG electrodes which penetrated at a 1 mm depth at the abdominal muscle. A ground electrode (3 M^TM^ Red Dot^TM^ resting EKG Diagnostic Electrode; 3 M, London, Ontario, Canada) was positioned on the mouse tail. Mice were securely restrained in a rodent sling (Lomir Biomedical, Notre Dame de-l’île-Perrot, Canada), and the electrodes and the balloon catheter were connected to a barostat to measure abdominal muscle contractions as an index of visceral sensitivity. After 10 min of rest, the balloon was progressively inflated with volumes of 100, 200 and 300 μL for 10 seconds, performed in triplicate, with resting intervals of 4 min, for a total duration of 36 minutes. For each distension, a 10 second baseline and 10-second stimulation periods were recorded. Electromyogram activity of the abdominal muscle was continuously recorded using customized software (Labview express 7.1).^39^ For stimulation experiments, CRD was performed 10 minutes after vehicle, capsaicin or GPCR agonists administration. The total area under the curve (AUC) was calculated, representing the sum of all data points across all three distension volumes.

## Primary culture of dorsal root ganglia (DRG) neurons

After mouse sacrifice, DRG were rinsed in HBSS and incubated in 6 mL of L-cysteine (Sigma-Aldrich, Oakville, Canada) containing 0.1 mg/mL of papain (Sigma-Aldrich) for 10 min at 37°C. After a wash with Leibovitz’s L-15 medium containing 10% Fetal Bovine Serum (FBS) (Gibco, Grand Island, US) and a wash with HBSS, DRG were incubated in 5 mL of HBSS with collagenase (Sigma-Aldrich) 1 mg/mL and dispase II (Sigma-Aldrich) 4 mg/mL for 2 times 5 min at 37°C. DRG were dissociated mechanically between the two incubation periods. Leibovitz’s L-15 medium was added to block enzymatic activities. DRG were then centrifuged (65 g, 5 min, 22°C) and re-suspended in Dulbecco’s modified Eagle’s medium (Gibco) containing 3% FBS, 1% of penicillin 100 U.mL^−1^/streptomycin 100 mg.mL^−1^ (Gibco), and 0.02% mitosis inhibitor: 5-Fluoro-2’-deoxyuridine, uridine, cytosine arabinoside, 10 μM each (Sigma-Aldrich). Finally, 200 μL of the DRG neurons in suspension were cultured in LabTek II Nunc®-CC2 ™ Chamber Slide ™ system (ThermoFisher, Mississauga, Canada) and incubated at 37°C in a 5% atmosphere of CO_2_ for 16–24 hours.

## Calcium imaging of DRG neurons

After 16–24 hours of culture, DRG neurons were incubated with HBSS containing 20 mM HEPES, 1 mM fluo-4 acetoxymethyl (AM) diluted in 5 µL of 20% pluronic F-127 and 45 µL of dimethylsulfoxide (DMSO) for 30 minutes at 37°C and 30 minutes in the dark at room temperature. At the end of the incubation time, HHBSS-Fluo-4AM was replaced by 100 μL of HBSS containing Ca^2+^ and Mg^2+^ per well. The change in dynamics in fluorescence intensity (ΔF/F) of Fluo-4AM was measured using an imaging system (Leica, DMI4000 inverted microscope, Canada) with a 10× objective and a kinetic of 80 recordings (one per second) was performed. After 5 seconds of baseline recording, the DRG neurons were exposed to either capsaicin (12.5 nM, 125 nM and 1250 nM), GPCR mix agonists (histamine, serotonin, bradykinin; 0.3 µM, 3 µM and 30 µM) (Sigma Aldrich) or vehicle (HBSS) (Sigma Aldrich). After 60 seconds of acquisition, a depolarizing concentration of potassium chloride (KCl, 50 mM) was added to discriminate sensory neurons from non-neuronal cells. The acquired fluorescence data were analyzed using ImageJ software to determine the fluorescence intensity of each cell (ΔF/F) and the percentage of responding neurons.

## Quantification of SP and CGRP production by DRG neurons

Dorsal root neurons were cultured as described above, except that 200 µL of medium containing sensory neurons were cultured in a 24-well plate previously coated with 10 µg/mL of laminin (L2020, Sigma Aldrich) and 50 µg/mL of poly-D-Lysine (P7280, Sigma-Aldrich). DRG neurons from 2 mice/6 wells were used. After 48 hours, the neuronal culture medium was replaced by 200 µl of fresh Dulbecco’s modified Eagle’s medium, and 25 µL of protease inhibitor cocktail (P2714, Sigma Aldrich), containing 2 mM of AEBSF, 0.3 µM of aprotinin, 116 µM of bestatin, 14 µM of E64, 1 µM of leupeptin and 1 mM of EDTA, were added to avoid substance P and CGRP degradation. DRG neurons were then stimulated with either vehicle (HBSS), capsaicin (1.25 µM) or GPCR agonists (30 μM) for 1 hour at 37°C with 5% CO_2_, the supernatants were then collected and immediately frozen at −80°C. Neuronal supernatants were tested with commercial ELISA kits for Substance P (Enzo, Burlington, Ontario) and CGRP (EIA kit, Bertin technologies, Montigny-le-Bretonneux, France) following the manufacturer’s recommendations.

## Statistical analysis

Statistical analyses were performed using GraphPad Prism 9. The data are presented either as mean ±SEM, or median (IQR). Statistical comparisons were performed using unpaired two-tailed t-test, Kruskal-Wallis or 2-way ANOVA, as appropriate. When multiple comparisons were performed, Dunn’s tests was used for Kruskal-Wallis and Šidak’s test was used for 2-way ANOVA. *P* < 0.05 was considered statistically significant.

To highlight the respective roles of the microbiota and sex, we have chosen two approaches how to present our data. First, considering the presence of microbiota as the main factor, we analyzed the data as pooled, as well as separately in males and females, and presented them in the main figures. The supplementary data then highlight sex as the main factor and are presented as pooled, as well as separately in germ-free and SPF mice.

## Results

### Gut microbiota influences visceral sensitivity in vivo in both female and male mice

**Figure 1.**
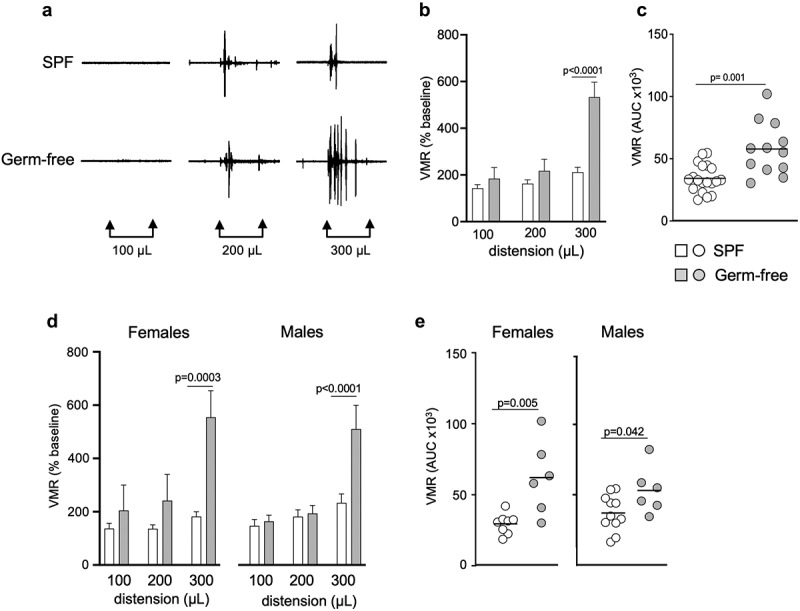
Gut microbiota modulates visceral sensitivity *in vivo* in both sexes. (a) Representative traces of visceromotor responses (VMR) to isovolumic colorectal distension (CRD) at 100 μL, 200 μL and 300 μL in conscious SPF and germ-free (GF) mice. (b) Basal state VMR of SPF (*n* = 19) and GF (*n* = 12) mice to CRD at 100 μL, 200 μL, 300 μL. VMR is expressed as % of baseline (white bar: SPF; gray bar: GF). (c) Total area under the curve (AUC) of the VMR to CRD in SPF and GF mice. (d) Basal state VMR of SPF female (*n* = 8) and male (*n* = 11), and GF female (*n* = 6) and male (*n* = 6) mice to CRD. (Ee) AUC of the VMR to CRD in female and male SPF and GF mice. White bar/circle: SPF; gray bar/circle: GF. Data are represented as mean ± SEM (**b**) (**d**), scatter dot plot with mean (**c) (e)**. Statistical analysis was performed using Mann-Whitney t-test **(c) (e)** and 2-way ANOVA followed by šidak’s multiple comparisons test (**b**) (**d**).

Visceral sensitivity in conscious mice was assessed by visceromotor responses (VMR) to isovolumic colorectal distensions (CRD). We found that germ-free mice displayed greater responses to CRD for the distension volume of 300 μL (*p* < 0.0001), as well as greater combined responses as calculated by the total area under the curve (AUC, *p* = 0.001) compared to SPF mice (Figure 1a,b,c). This increased visceral sensitivity was observed in both germ-free male and female mice (Figure 1d,e). There was no difference in colonic compliance between germ-free and SPF mice (Fig. S1a). This suggests that the gut microbiota modulates visceral sensitivity regardless of sex, and that the increased visceral sensitivity observed in germ-free mice is not due to the mechanical properties of the colon.

To assess whether sex *per se* could have an influence on pain perception in physiological conditions, we compared CRD responses in both SPF and germ-free conditions and found that there were no differences in responses to CRD between males and females (Fig. S2a). These data indicate that, at basal (unstimulated) state, females and males exhibit similar pain sensitivity.

## Gut microbiota affects TRPV1 signaling in female mice

**Figure 2.**
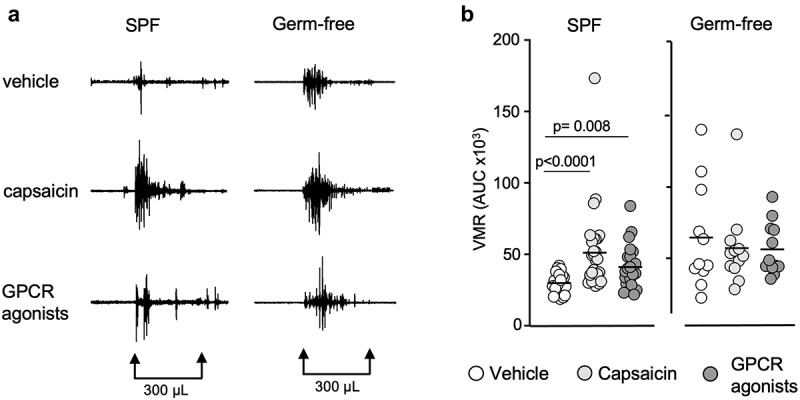
Capsaicin and GPCR agonists induce visceral hypersensitivity in SPF mice but not in germ-free mice. (a) Representative traces of VMR to CRD at 300 μL in conscious SPF and germ-free (GF) mice in response to vehicle, capsaicin (30 μg) or GPCR agonists (30 μg). (b) AUC of the VMR after vehicle, capsaicin or GPCR agonists administered intracolonically (white circle: vehicle; light gray circle: capsaicin; dark gray circle: GPCR agonists). Data are represented as scatter dot plot with means **(b)**. Statistical analysis was performed using Kruskal-Wallis followed by Dunn’s post hoc test.

**Figure 3.**
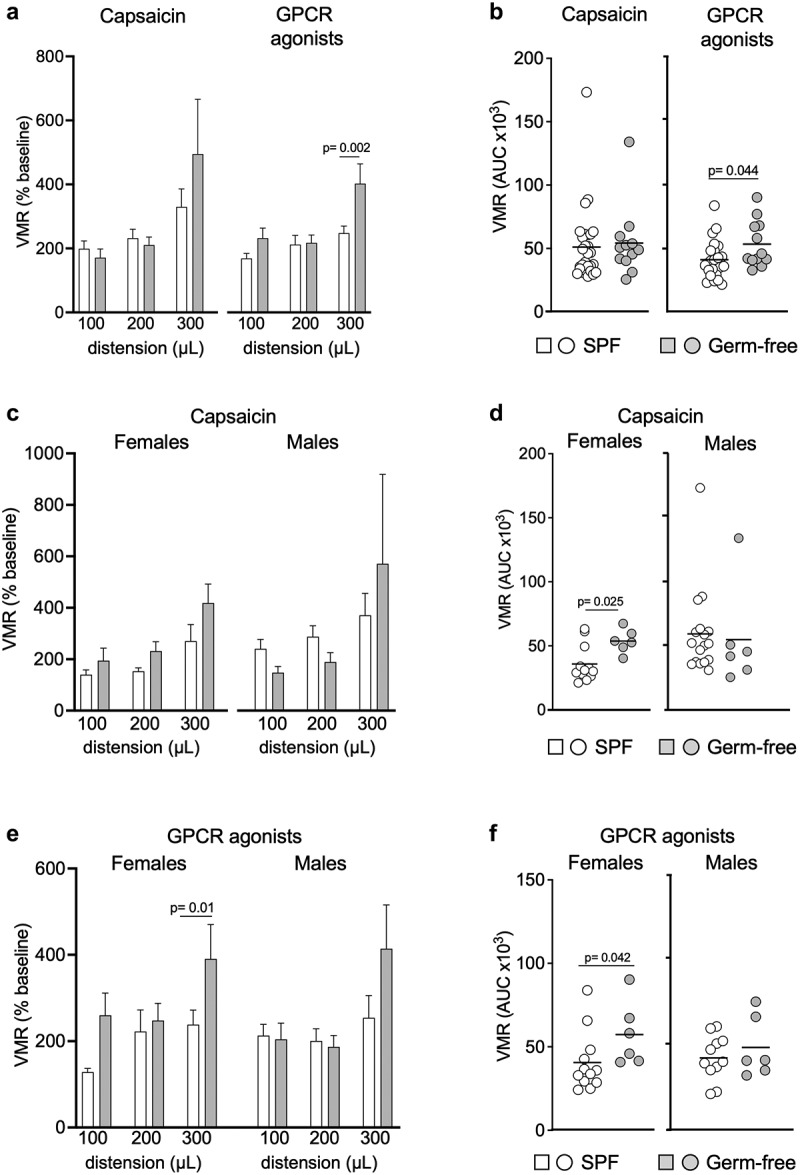
GPCR agonists and capsaicin pre-treatment induce greater responses to CRD in germ-free female mice. (a) VMR of SPF (*n* = 29) and GF (*n* = 12) mice after intracolonic administration of capsaicin or GPCR agonists at distensions of 100 μL, 200 μL and 300 μL. VMR is expressed as % of baseline (white bar: SPF; gray bar: GF). (b) AUC of the VMR after intracolonic administration of capsaicin or GPCR agonists in SPF and GF (white circle: SPF; gray circle: GF). (c) VMR of SPF female (*n* = 12) and male (*n* = 17) and GF female and male (each *n* = 6) after administration of capsaicin. (d) AUC of the VMR after capsaicin intracolonic administration in SPF and GF female and male. (e) VMR of SPF female (*n* = 12) and male (*n* = 11) and GF female and male (both *n* = 6) after intracolonic administration of GPCR agonists. (f) AUC of the VMR after administration of GPCR agonists in SPF and GF. White bar/circle: SPF; gray bar/circle: GF. Data are represented as means±sem (a) (c) (e), scatter dot plot with means (b) (d) (f). Statistical analysis was performed using 2-way ANOVA followed by šidak’s multiple comparisons test (a) (c) (e) and Mann-Whitney t-test (b) (d) (f).

Capsaicin, the active component of the chili pepper, is known to activate the transient receptor potential vanilloid 1 (TRPV1), sensitize peripheral sensory nerve fibers and induce gut nociception.^40^ To investigate whether the gut microbiota affects responses to capsaicin, we administered intracolonic capsaicin (30 μg/mouse) prior to CRDs. As expected, capsaicin increased responses in SPF mice compared to the vehicle, however this increase was not observed in germ-free mice (Figure 2a,b). This difference was due to higher responses to vehicle found in germ-free mice, as VMRs in SPF and germ-free mice were similar after intracolonic instillation of capsaicin (Figure 3a,b). When considering sex in our analysis, we found opposite trends in males and females. While VMRs for the higher distension volumes were higher in germ-free females compared to SPF females (*p* = 0.025) (Figure 3c,d), responses in germ-free males appeared lower compared to their SPF counterparts (Figure 3c,d), although this did not reach statistical significance. Similar to baseline experiments, there was no difference in colonic compliance after capsaicin administration between SPF and germ-free mice (Fig. S1b).

When stratifying results primarily by sex, we found that capsaicin-stimulated responses to CRD were higher in SPF males compared to SPF females (Fig. S2b), but this was not true in germ-free conditions. Overall, these data suggest that the gut microbiota affects capsaicin-induced visceral hypersensitivity and that microbiota-sex interactions alter the response to capsaicin.

## Gut microbiota affects GPCR signaling

We then investigated whether GPCR activation by mixed agonists (histamine, bradykinin and serotonin), which induce peripheral sensitization of nerve fibers during inflammation,^8,41–43^ is affected by the gut microbiota. Intracolonic instillation of GPCR agonists (30 μg/mouse) increased responses to CRD in SPF mice compared to the vehicle, but this was not observed in germ-free mice (Figure 2a,b), with no difference in colonic compliance noted (Fig. S1c). The GPCR agonists-stimulated responses were greater in germ-free mice for the distension volume of 300 μL (*p* = 0.002) (Figure 3a) and the total AUC (*p* = 0.044) (Figure 3b) than SPF mice, and this was driven by higher responses in germ-free females, as responses in males were similar (Figures 3e,3f). These results suggest that GPCR signaling might be involved in the increased visceral sensitivity observed in germ-free mice, mainly in females. Sex primarily, however, did not influence responses to GPCR activation, as we observed no differences between female and male mice, when compared either in SPF or germ-free conditions (Fig. S2c).

## Gut microbiota and sex do not influence DRG neuron activity

**Figure 4.**
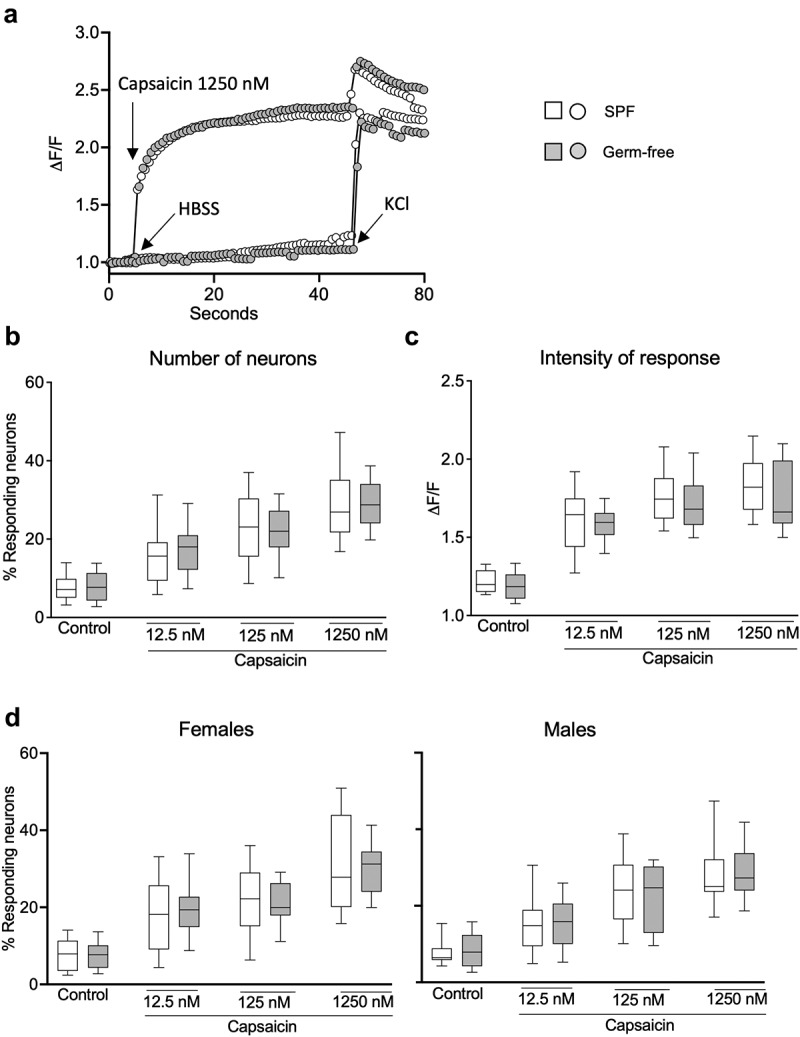
DRG neuronal activation is similar in SPF and GF mice after TRPV1 activation. (a) Representative fluorescent traces of calcium flux in DRG neurons from SPF and GF mice in response to vehicle (HBSS) or capsaicin (1250 nm). (b) Percentage of responding DRG neurons obtained from SPF (white box) and GF (gray box) mice, after treatment with capsaicin (12.5 nM, 125 nM, 1250 nM). (c) Intensity of the neuronal response (ΔF/F) in DRG neurons obtained from SPF and GF mice, after treatment with capsaicin. (d) Percentage of responding neurons obtained from SPF females and males and from GF female and male mice, after treatment with capsaicin. White box: SPF, gray box: GF. Data are represented as box plots (10–90%ile) with *n* = 8 independent experiments of 1–2 wells per condition for SPF females; *n* = 7 independent experiments of 1–2 wells per condition for SPF males; *n* = 5 independent experiments for both GF females and males mice. In each well, 20–130 neurons were cultured. Statistical analysis was performed using 2-way ANOVA followed by šidak’s multiple comparisons test.

To investigate whether gut microbiota affects peripheral sensory signaling, we performed calcium mobilization studies using primary culture of DRG neurons obtained from SPF and germ-free mice. Compared with the vehicle HBSS, exposure to capsaicin (1250 nM) induced a typical TRPV1 activation accompanied by a plateau phase in neurons obtained both from SPF and GF mice (Figure 4a). Treatment with capsaicin (12,5 nM, 125 nM and 1250 nM) increased calcium flux in a dose-dependent fashion similarly in SPF and germ-free mice, as assessed both by a higher percentage of responding neurons and intensity of neuronal response (ΔF/F), compared to the vehicle HBSS (Figure 4b-d). Capsaicin-stimulated DRG neuronal activity was similar between SPF and germ-free mice, with no additional effect of sex (Figure 4b-d).

**Figure 5.**
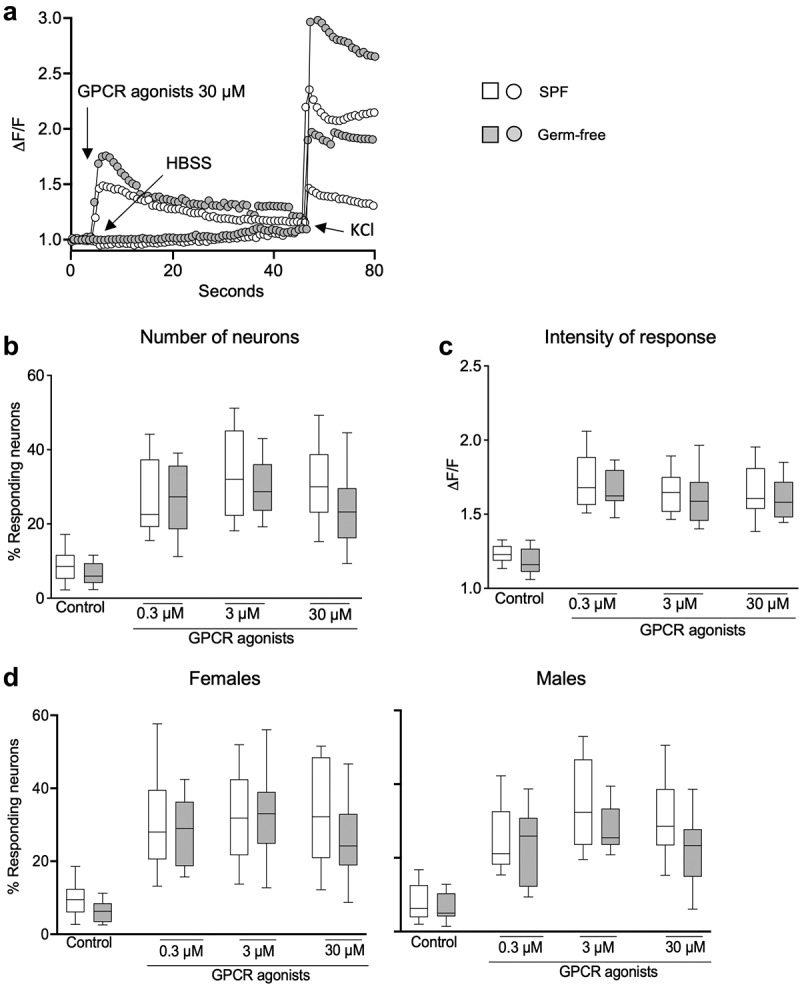
DRG neuronal activation is similar in SPF and GF mice after GPCR activation. (a) Representative fluorescent traces of calcium flux in DRG neurons from SPF and GF mice in response to vehicle (HBSS) or GPCR agonists (30 μM). (b) Percentage of responding DRG neurons obtained from SPF (white box) and GF (gray box) mice, after treatment with GPCR agonists (0.3 μM, 3 μM, 30 μM). (c) Intensity of the neuronal response (ΔF/F) in DRG neurons obtained from SPF and GF mice, after treatment with GPCR agonists. (d) Percentage of responding neurons obtained from SPF females and males and from GF female and male mice, after treatment with capsaicin. White box: SPF, gray box: GF. Data are represented as box plots (10–90%ile) with *n* = 8 independent experiments of 1–2 wells per condition for SPF females; *n* = 7 independent experiments of 1–2 wells per condition for SPF males; *n* = 5 independent experiments for both GF females and males mice. In each well, 20–130 neurons were cultured. Statistical analysis was performed using 2-way ANOVA followed by šidak’s multiple comparisons test.

Exposure of DRG neurons to GPCR agonists (30 μM) induced a typical GPCR activation with a bell-shaped curve in both SPF and GF mice compared with the vehicle HBSS (Figure 5a). Administration of GPCR agonists (0.3 μM, 3 μM, 30 μM) increased the percentage of responding neurons (Figure 5a) and ΔF/F in both SPF and germ-free mice, compared to vehicle (Figure 5b). There was, however, no difference in the GPCR-induced neuronal activity between SPF and germ-free mice (Figure 5c), with sex having no additional effect.

We further explored the influence of sex on neuronal activity and found that stimulation with either vehicle, capsaicin or GPCR agonists induced similar responses in DRG neurons obtained from male and female mice (Fig. S3a, b). Thus, these data suggest that nociceptors activation in DRG neurons is neither microbiota nor sex-dependent.

## The gut microbiota influences production of CGRP but not SP in DRG

**Figure 6.**
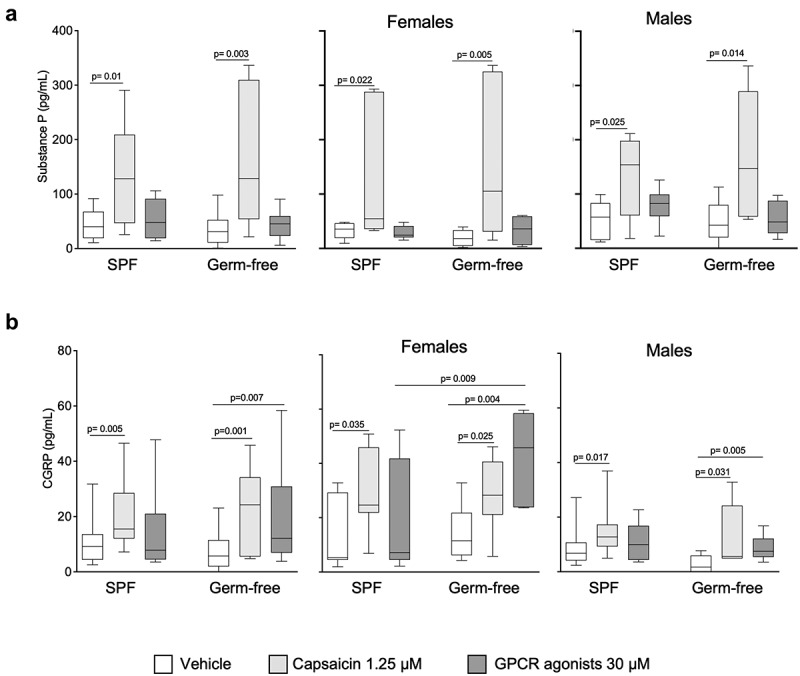
CGRP, but not substance P production by DRG neurons is higher in GF females after GPCR activation. (a) SPproduction in DRG neurons cultured *ex vivo* from SPF and GF mice of both sexes in response to vehicle (HBSS), capsaicin (1.25 μM) or GPCR agonists (30 μM). Data are expressed as box plots (10–90%ile) with *n* = 3 independent experiments of 1–4 wells per condition for SPF male/SPF female mice and GF male/GF female mice. (b) CGRP production in DRG neurons cultured *ex vivo* from SPF and GF both sexes in response to vehicle (HBSS), capsaicin (1.25 μM) and GPCR agonists (30 μM). Data are expressed box plots (10–90%ile); *n* = 4 independent experiments of 2-wells per condition for SPF male; *n* = 3 independent experiments (*n* = 2–4) for SPF female, GF male and GF female mice. Statistical analysis was performed using Kruskal-Wallis followed by Dunn’s post hoc test.

To assess whether the gut microbiota affects the production of neurotransmitters involved in pain transmission, we focused on SP and CGRP as the key neuropeptides released by DRG neurons.^13^ Capsaicin, but not GPCR agonists, increased SP production in DRG neurons, with similar responses in those isolated from SPF and germ-free mice, or from males or females (Figure 6a). When stratifying results by sex, we found that the production of substance P after stimulation with vehicle or capsaicin was not sex-dependent. However, it was higher after the GPCR activation in male mice in SPF conditions. (Fig. S4a).

Capsaicin administration increased CGRP production in DRG neurons isolated from both SPF and GF mice (Figure 6b), irrespectively of sex. On the contrary, GPCR agonists increased CGRP production only in DRG neurons isolated from germ-free mice, and this was mainly driven by responses in females (Figure 6b), which were also higher than in SPF females.

When stratifying primarily by sex, CGRP production in response to capsaicin was greater in females than males, in both SPF and germ-free mice (Fig. S4b). Moreover, its production after vehicle or GPCR agonists stimulation was higher in females in germ-free mice (Fig. S4b). Overall, these data suggest that substance P is largely unaffected by microbiota or sex, while the CGRP production appears to be dependent both on the presence of microbiota and sex.

## Discussion

Gut microbiota has been suggested to be a key player in determining gut function, including visceral sensitivity, by interacting with the enteric and central nervous system or by modulating the intestinal immune responses.^44,45^ A previous study demonstrated that visceral sensitivity is increased in male germ-free mice and that it normalizes after bacterial colonization, associated with changes in the volumes of the anterior cingulate cortex and periaqueductal gray, two brain areas involved in pain processing.^16^ However, a subsequent study in female mice failed to show any difference between germ-free and conventional mice, and visceral pain perception was suggested to be estrous cycle-dependent only in the latter ones.^28^ Together, these results suggested that central neural mechanisms may underly altered pain perception in germ-free mice, and that sex differences have a major role in their expression.

In our study we investigated the peripheral pain mechanisms at the level of the gut and primary sensory neurons, in the presence or absence of gut microbiota. First, we found that visceral mechanosensitivity is higher in germ-free mice, in both males and females. Moreover, colon compliance is similar between germ-free and conventional mice, thus ruling out mechanical properties of the colon as a factor involved in increased sensitivity observed in germ-free mice.

Second, we discovered that DRG neuronal activity, assessed by calcium imaging after capsaicin or GPCR agonists stimulation, is similar between germ-free and conventional mice, with no difference between males and females. This suggests that the gut microbiota does not affect peripheral neuronal activation. Although several studies investigated the influence of gut microbiota on the brain and the spinal cord, assessing anxiety-like behavior^46–49^ and pain-related behaviors,^16^ no studies explored the influence of gut microbiota in visceral pain in periphery. Our study thus provides novel insights into the peripheral pain mechanism by focusing on the influence of gut microbiota on DRG sensory neurons.

Third, we assessed the production of SP and CGRP by DRG neurons, as key neuropeptides involved in the mediation of the nociceptive messages from the periphery to the brain.^50^ We found that the production of substance P in response to vehicle, capsaicin or GPCR agonists was similar in DRG neurons obtained from germ-free and conventional mice. Although previous studies demonstrated changes in SP levels induced with *Lactobacillus paracasei*^17^ or bacterial toxins,^51^ our study shows that the conventional healthy (SPF) microbiota does not affect the production of SP in primary sensory neurons. We have, however, found that DRG neurons from germ-free mice, when stimulated by GPCR agonists, produce more CGRP, and this is more pronounced in females. Interestingly, visceral mechanosensitivity after GPCR agonists pre-treatment is higher in germ-free females, which could be in part explained by the higher CGRP production by the DRG neurons.

We found that visceral sensitivity is higher in GF female compared to SPF female mice after intracolonic administration of capsaicin but similar in male mice. In line with our results obtained in males, it has been shown that the stimulation of colon mucosa-submucosa preparations with capsaicin-induced similar neural activity in germ-free and SPF mice^52^ and that TRPV1 mRNA expression in the spinal cord was comparable between SPF and germ-free male mice.^16^ In contrast, capsaicin-induced pain behaviors were lower in antibiotic-treated mice compared to vehicle-treated mice of both sexes^53,,54^ suggesting that pain responses in antibiotic-treated and germ-free mice may differ.

It should be noted, however, that overall the pre-treatment with both capsaicin and GPCR agonists increased more responses in SPF than in germ-free mice, which may be due to lower intestinal permeability in the latter,^55^ reducing access of capsaicin and GPCR agonists to sensory neurons.

The substantial core of clinical literature indicates that women experience increased visceral sensitivity and higher risk for pain than men,^56^ although some studies contradict this concept.^29,35^ The mechanisms of action involved in the development of pain vary in males and females. In males, pain perception has been suggested to depend on microglia and the TLR4 receptor present in the spinal cord, whereas in females, pain may be induced by the adaptive immunity, mainly T lymphocytes present in the spinal cord.^29,,30^ We show that in basal (unstimulated) conditions or after intracolonic administration of GPCR agonists, the pain sensation in male and female mice, in SPF or germ-free conditions, is similar. Indeed, it has been shown that histamine produces a clear axon reflex flare due to an increase of skin blood flow similarly in both males and females.^57^ In contrast, we show that intracolonic administration of capsaicin-induced overall higher responses in SPF males compared to SPF female mice, in agreement with a recent study demonstrating sexual dimorphism in behavioral manifestation of capsaicin-induced hypersensitivity, observing an increase in abdominal contraction only in male mice.^31^ Although DRG neuronal activation in response to capsaicin in our study is similar in males and females, a previous electrophysiology study showed that capsaicin-induced inward currents in DRG neurons of male mice were considerably greater than in female mice, which was associated with a higher phosphorylated TRPV1 protein.^58^ In parallel, we show that the GPCR agonists-induced production of SP is higher in SPF male than female mice. However, in germ-free conditions, the production of CGRP by DRG neurons is greater in females compared to male mice. Several previous studies showed that females have higher mechanical sensitivity than males in response to CGRP in migraine pain models,^59,,60^ highlighting a female-specific role of CGRP in pain perception. Our study thus provides further evidence of the existence of sex differences in visceral pain sensation, which is, at least in part, mediated by peripheral mechanisms.

Our study has several weaknesses, as we did not access the estrous cycle and thus we cannot exclude that the increase in visceral sensitivity observed in germ-free females is linked to the female hormonal changes.^28^ Moreover, we did not evaluate the intestinal permeability between conventional and germ-free mice, and therefore we cannot rule out different access of intracolonic capsaicin and GPCR agonists to DRG neurons. Importantly, we used a mixture of GPCR agonists (histamine, serotonin and bradykinin) known to be involved in pain and inflammation, which act through the largest and most functionally diverse family of receptor, that can both stimulate and inhibit pain transmission.^61^ We have chosen this approach as it was previously used to screen for microbiota-mediated effects on nociception^26,,38^ and because these agonists have been associated with gut microbiota. Serotonin production in the intestine was shown to be affected by the gut bacteria^62^ and bacterial histamine can trigger visceral hyperalgesia through H4 receptor pathways.^39^ However, in order to get a full picture of the role of microbiota in GPCR-mediated pain signaling, individual agonists and different subtypes of receptors should be investigated in future studies.

Taken all together, our study provides new insights into the peripheral regulation of visceral pain demonstrating that visceral sensitivity is modulated both by the microbiota status and sex. Germ-free mice, both males and females, have greater *in vivo* responses to colorectal distension than conventional mice, but the stimulated responses with capsaicin and GPCR agonists are higher in germ-free females only. However, at the level of DRG neurons, neither the gut microbiota nor sex affect the neuronal activation or production of SP. Finally, we show that the CGRP production in the DRG neurons is greater in germ-free mice, with levels being higher in female mice, which may underlie the increased *in vivo* responses to colorectal distension observed in mice raised without gut microbiota.

## Supplementary Material

Supplemental Material

## Data Availability

The main and supplementary data that support the findings of this study are available in figshare at https://doi.org/10.6084/m9.figshare.21430500
