# Supplementary material for: Gut microbiota modulates visceral sensitivity through calcitonin gene-related peptide (CGRP) production
Source: Gut Microbes. 2023 Mar 20;15(1):2188874. doi: 10.1080/19490976.2023.2188874 (PMC10038053; doi:10.1080/19490976.2023.2188874)
Supplement: Supplemental Material [file KGMI_A_2188874_SM0607.zip › microbiome_and_pain_Supplementary_figure_legend.docx]

**Supplementary figures legends**:

**Fig S1: Colonic compliance is similar between SPF and GF mice in both sexes in basal state and stimulated conditions**

**(A)** Colonic compliance (pressure in mmHg *vs* distension volume in μL) in SPF and GF mice in basal state. White circle: SPF; grey circle: GF

**(B)** Colonic compliance in SPF and GF mice in response to intracolonic administration of capsaicin (30 μg). White circle: SPF; grey circle: GF

**(C)** Colonic compliance in SPF and GF mice in response to intracolonic administration of GPCR agonists (30 μg). White circle: SPF; grey circle: GF

Statistical analysis was performed using two-way ANOVA and subsequent Šidak’s multiple comparisons test **(A)(B)(C)**.

**Fig S2: Visceral sensitivity is increased in SPF male mice in response to capsaicin but similar in males and females in basal state and after GPCR agonists administration.**

**(A)** AUC of the VMR to CRD in female and male SPF and GF mice in basal state. SPF female = 8; SPF male =11 and GF female= 6 mice GF male = 6. White circle: females; grey circle: males

**(B)** AUC of the VMR to CRD in female and male SPF and GF mice in response to intracolonic administration of capsaicin (30 μg). SPF female = 12; SPF male =17; GF male = 6; GF female mice =6. White circle: females; grey circle: males

**(C)** AUC of the VMR to CRD in female and male SPF and GF mice in response to intracolonic administration of GPCR (30 μg). SPF female = 12; SPF male =11; GF male = 6; GF female mice =6. White circle: females; grey circle: males

Data are represented as scatter dot plot mean. Statistical analysis was performed using Mann-Whitney t-test **(A)(B)(C)**.

**Fig S3**: **Neuronal activity is similar in male and female SPF and GF mice**

**(A)** Percentage of responding neurons in SPF and GF female and in SPF and GF male DRG neurons after treatment with capsaicin (12.5 nM, 125 nM and 1250 nM). White box: females; grey box: males

**(B)** Percentage of responding neurons in SPF and GF female and in SPF and GF male DRG neurons after treatment with the GPCR agonists (0.3 μM, 3 μM, 30 μM). White box: females; grey box: males.

Data are represented as box and whiskers 10-90 percentile n= 7 independent experiments of 1-2 wells per condition for SPF male; n=8 independent experiments of 1-2 wells per condition for SPF female in response to capsaicin and GPCR agonists. n= 5 independent experiments for both GF females and males mice in response to capsaicin and GPCR agonists. In each well, 20-130 neurons were cultured. Statistical analysis was performed using two-way ANOVA and subsequent Šidak’s multiple comparisons test

**Fig. S4: CGRP production is higher in germ-free female mice**

(**A**) Substance P production in DRG neurons cultured *ex vivo* from SPF and GF male and SPF and GF female mice in response to vehicle (HBSS), capsaicin (1250 nM) or GPCR agonists (30 μM). White box: females; grey box: males.

Data are expressed as box and whiskers 10-90 percentile; n=3 independent experiments of 1-4 wells per condition for SPF male/SPF female mice and GF male/GF female mice. Statistical analysis was performed using a Mann-Whitney test.

(**B**) CGRP production in DRG neurons cultured *ex vivo* from SPF and GF male and SPF and GF female mice in response to vehicle (HBSS), capsaicin (1250 nM) or GPCR agonists (30 μM). Data are expressed as box and whiskers 10-90 percentile; n=4 independent experiments of 2-4 wells per condition for SPF male; n=3 independent experiments (n=2-4) for SPF female; n=3 independent experiments (n=2-4) for GF male and GF female mice.

Statistical analysis was performed using a Mann-Whitney test.
