# Supplementary figures and images for: Gut microbiota modulates visceral sensitivity through calcitonin gene-related peptide (CGRP) production
Source: Gut Microbes. 2023 Mar 20;15(1):2188874. doi: 10.1080/19490976.2023.2188874 (PMC10038053; doi:10.1080/19490976.2023.2188874)

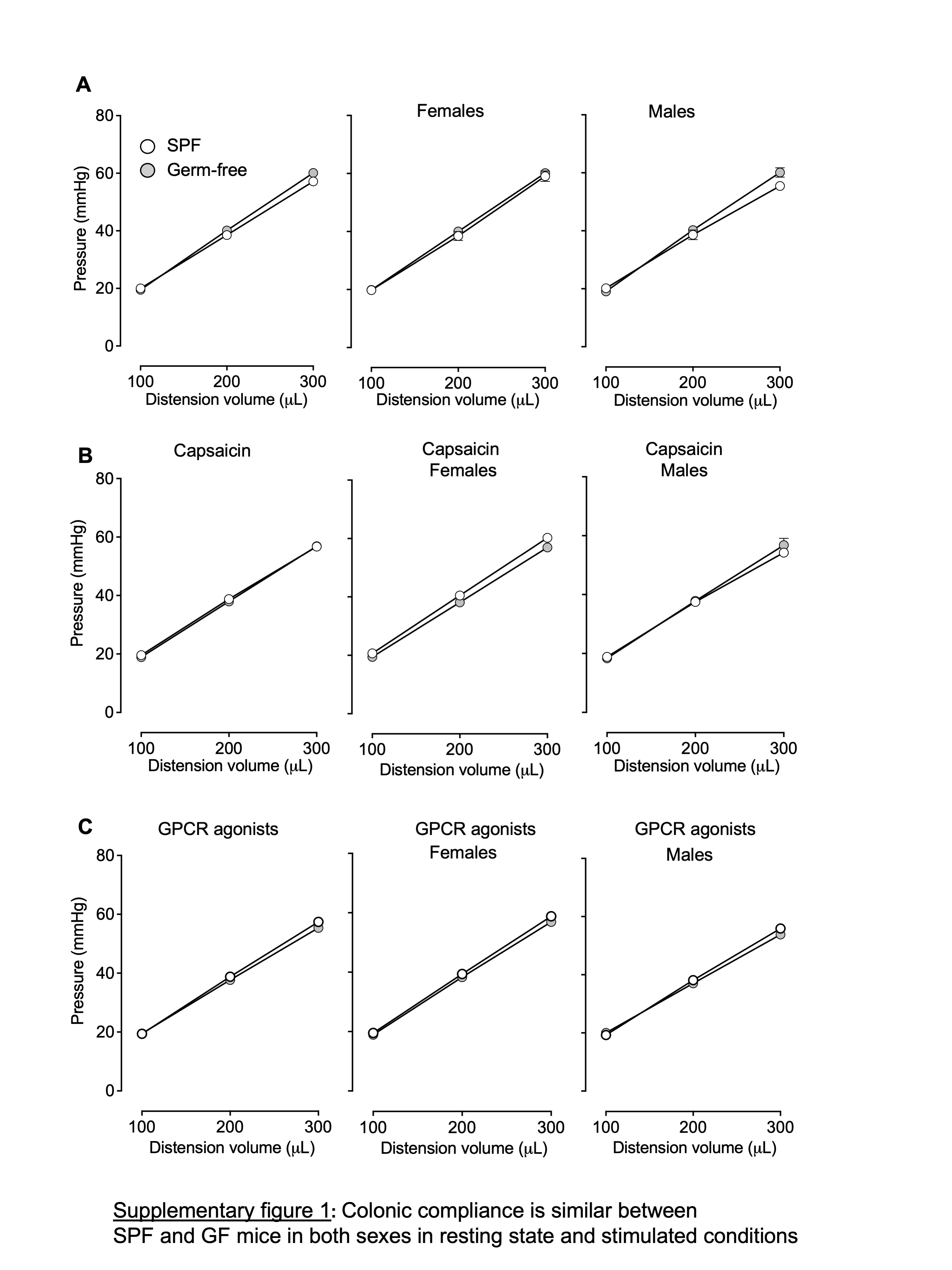

Supplement: Supplemental Material [file KGMI_A_2188874_SM0607.zip › Supplementary figure 1.tiff]

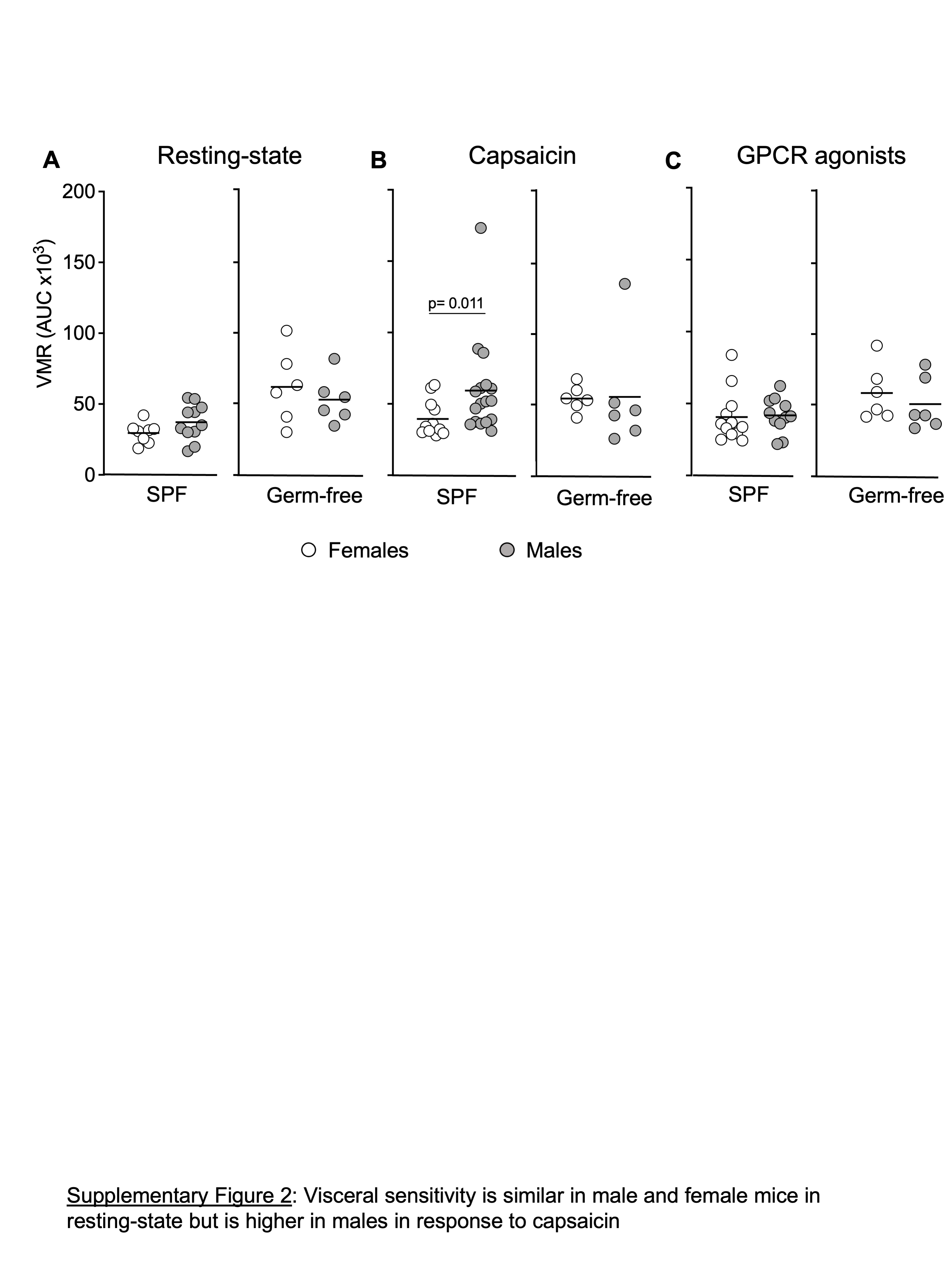

Supplement: Supplemental Material [file KGMI_A_2188874_SM0607.zip › Supplementary figure 2.tiff]

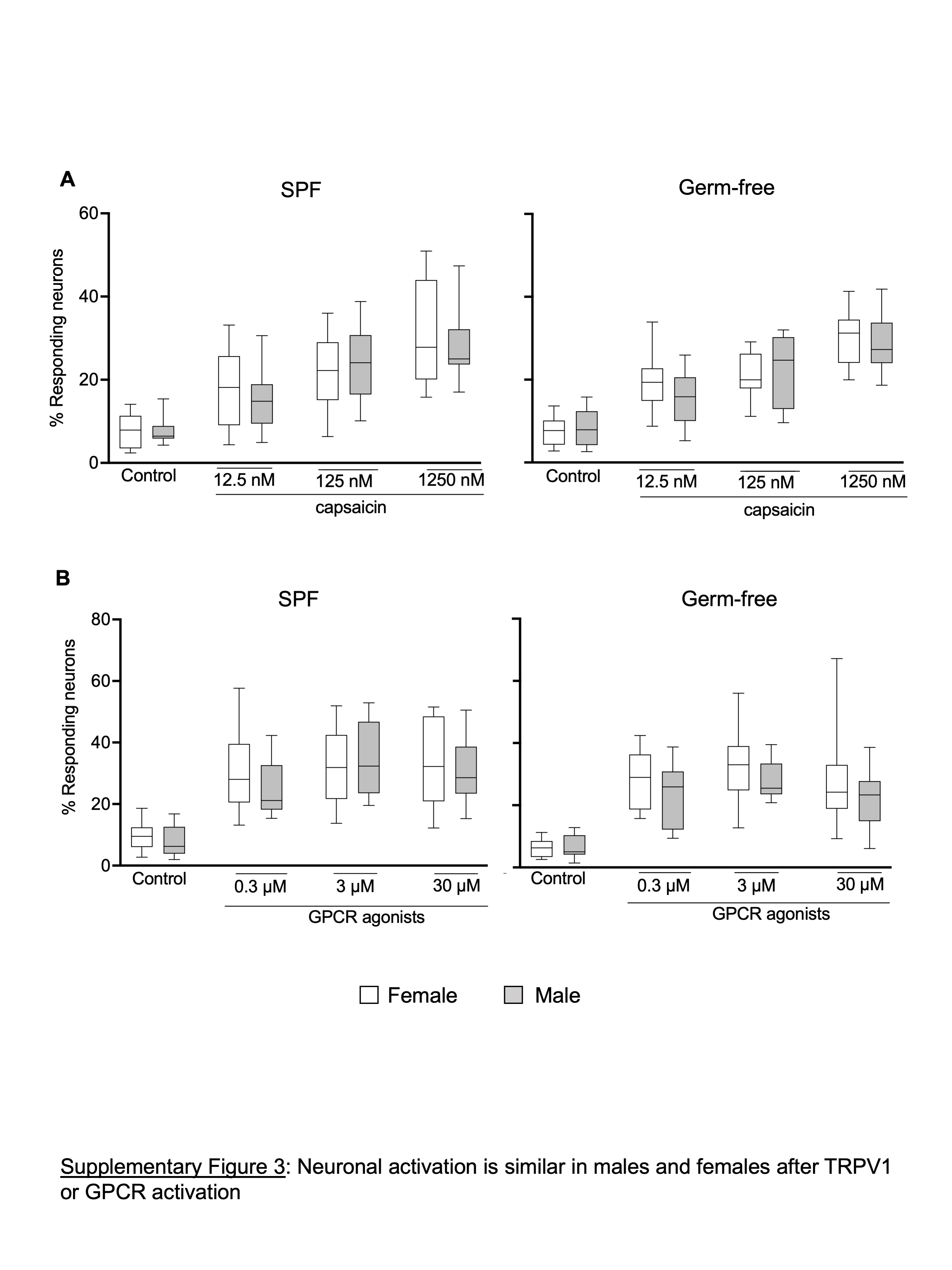

Supplement: Supplemental Material [file KGMI_A_2188874_SM0607.zip › Supplementary figure 3.tiff]

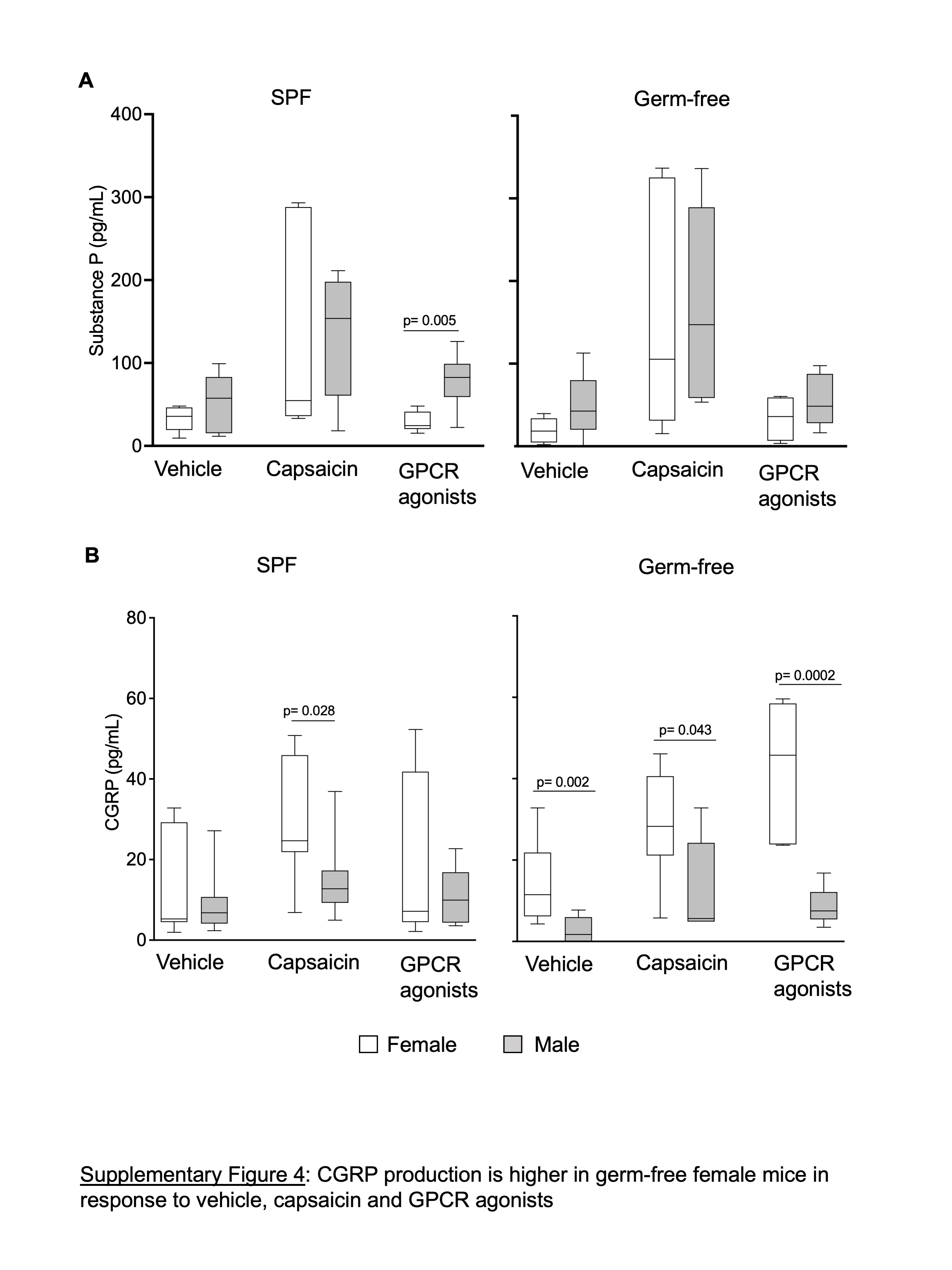

Supplement: Supplemental Material [file KGMI_A_2188874_SM0607.zip › Supplementary figure 4.tiff]
